# Apoptosis Induction of *Salvia chorassanica* Root Extract on Human Cervical Cancer Cell Line

**Published:** 2013

**Authors:** Heydar Parsaee, Javad Asili, Seyed Hadi Mousavi, Hojjat Soofi, Seyed Ahmad Emami, Zahra Tayarani-Najaran

**Affiliations:** a *Department of Pharmacology and Pharmacological Research Center of Medicinal Plants, School of Medicine, Mashhad University of Medical Sciences, Mashhad, Iran.*; b *Department of Pharmacognosy, School of Pharmacy, Mashhad University of Medical Sciences, Mashhad, Iran.*; c *Medical Toxicology Research Center, Mashhad University of Medical Sciences, Mashhad, Iran.*; d *Research Center of Natural Products Safety and Medicinal Plants, North Khorasan University of Medical Sciences, Bojnurd, 01830-49504, Iran. *; e *Department of Pharmacodynamics and Toxicology, School of Pharmacy, Mashhad, University of Medical Sciences, Mashhad, Iran. *

**Keywords:** Apoptosis, *Salvia chorassanica*, Lamiaceae, Caspase

## Abstract

*Salvia chorassanica *Bunge is one of the Iranian endemic species of *Salvia*. There is not any reported literature on *S. chorassanica. *This study was designed to examine the *in-vitro *anti-proliferative and proapoptotic effects of the methanol extract of *S. chorassanica *and its fractions on HeLa cell line. Cells were cultured in EX-CELL®, an animal free medium specially designed for HeLa cell line and incubated with different concentrations of plant extracts. Cell viability was quantified by MTS assay. Apoptotic cells were determined using propidium iodide (PI) staining of DNA fragmentation by flow cytometry (sub-G1 peak). Activity of caspase -3, -8 and -9 was measured by the caspase colorimetric kit assay. *S. chorassanica *inhibited the growth of malignant cells and the CH_2_Cl_2_ fraction was determined as the most cytotoxic fraction in comparison with other fractions. The calculated IC_50_ values for methanol extract, *n*-hexane, CH_2_Cl_2_ and EtOAc fractions were 8.841, 5.45, 2.38, and 58.03 μg/mL, respectively. *S. chorassanica *induced a sub-G1 peak in the flow cytometry histogram of treated cells compared to control cells indicating that the cytotoxic mechanism is characterized by apoptosis induction. The activity of caspase-3 and 8 proteins in treated HeLa cells was significantly higher than that of the control while caspase-9 activity did not change significantly. Based on the result obtained from our study, the apoptosis pathway involved in *S. chorassanica-*induced cell death may be through the extrinsic pathway and it can be a novel promising candidate in the treatment of cancer.

## Introduction

Natural products have long been used as the important source of cancer treatment, which is estimated to become the major cause of death in the current century. There are more than one thousand species that have been found to possess significant anticancer properties (1). Several potential lead molecules have emerged as drugs upon modification of these natural leads and many more are yet to come (2). 

However, there is continues need for development of new anticancer drugs, by scientific exploration of a vast variety of synthetic, biological and natural products (1).

Clinical studies as well as experimental approaches have revealed the anticancer properties of a large amount of medicinal herbs that are mediated through different mechanisms including altered carcinogen metabolism, induction of DNA repair systems, immune activation and suppression of cell cycle progression/promotion of apoptosis (3).

Apoptosis is a gene-regulated phenomenon induced by many chemotherapeutic agents which is considered very useful in the management and therapy as well as in the prevention of cancer (4).

The genus *Salvia *(Lamiaceae) includes nearly 900 species spread throughout the world, 17 of which are endemic to Iran (5). *Salvia *species are known for various biological activities in folk and modern medicine.

Previous chemical investigations on different species of *Salvia *have shown the presence of flavonoids, diterpenoids, triterpenoids, sesterterpenes, and essential oils exhibits the antitumor, antimicrobial, cytotoxic and anti-inflammatory activities (6-8).

In Chinese traditional medicine, *S. miltiorrhiza *has been used for the treatment of menstrual disorder, menostasis, menorrhagia, insomnia, arthritis, and coronary heart diseases, particularly angina pectoris and myocardial infarction (9-11). In modern experimental studies, numerous diterpenoid tanshinones isolated from *S. miltiorrhiza *(12, 13) were shown to have various biological activities including antitumor (10, 14-16) and antimicrobial (17, 18) activities.

*S. chorassanica *Bunge is one of the Iranian endemic species of *Salvia *that only grows in Razavi Khorasan province. There is not any reported literature on *S. chorassanica. *However, different reports have verified the cytotoxic and antitumor properties of some species belonging to this genus. Therefore, this study was designed to explore the cytotoxic and proapoptotic effect of *S. chorassanica *on HeLa cell line. It has been one of the most widely studied cell lines in cervical cancer, the second most frequent malignant tumor in women worldwide (19). In this study, Paclitaxel was used as a positive control. EX-CELL^®^, an animal free medium was used in this study in all experiments in order to respect animal rights.

## Experimental

*Reagents and chemicals*

The fluorescent probe propidium iodide (PI), sodium citrate, Paclitaxel, EX-CELL® and Triton X-100 were purchased from Sigma (St Louis, MO, USA); 3-(4,5-dimethylthiazol-2-yl)-5-(3-carboxymethoxyphenyl)-2-(4-sulphophenyl)-2H-tetrazolium (MTS), from Promega (Madison, WI, USA); and caspase colorimetric kits from abcam (Cambridge, UK).

*Plant materials*

The roots of *S. chorassanica *were collected from Hosseinabad valley (2100 m height) in Pivejan, a village located in 65 km from south-west of Mashhad, Razavi Khorasan province, northeast of Iran. The plant was identified by Mr. M.R. Joharchi, from Ferdowsi University of Mashhad Herbarium (FUMH). Voucher specimen (No. 11289) was deposited in herbarium of faculty of pharmacy, Mashhad University of Medical Sciences.

*Cell culture*

HeLa cell line was obtained from Pasteur Institute (Tehran, Iran) and lymphocytes were isolated from human peripheral blood using the Lympholyte®-H (a density gradient separation medium) according to the manufacturer’s protocol and maintained at 37°C in a humidified atmosphere (90%) containing 5% CO_2_. Cells were cultured in EX-CELL^®^ medium, 100 U/mL penicillin and 100 mg/mL streptomycin. Cells were seeded overnight and then incubated with various concentrations of different extracts for 48 h. For MTS assay, cells were seeded at 5 × 10^4^ cell per well onto 96-well culture plates. For assay of apoptosis, cells were seeded at 10^5^ cells per well onto a 24-well plate.

For each concentration, there was a control sample that remained untreated and received the equal volume of medium.

*Extraction and fractionation*

**Figure 1:**
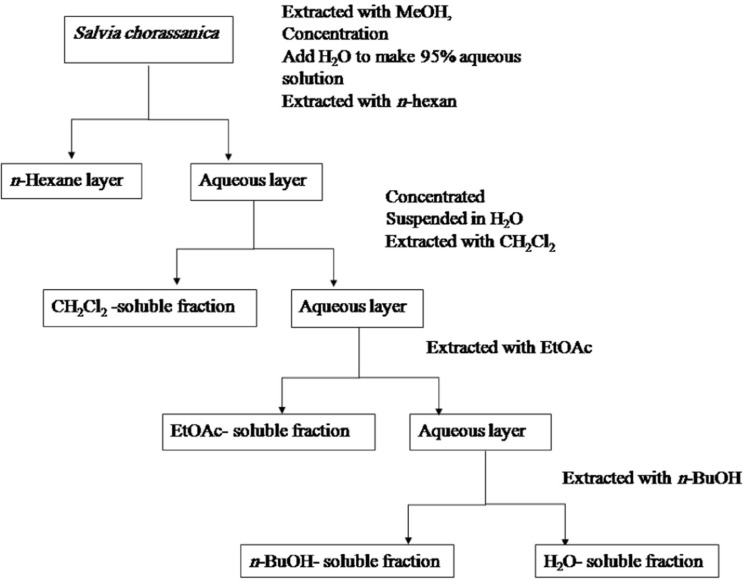
Partitioning scheme using immiscible solvents

The dried roots (100 g) were peculated with methanol at room temperature. The whole extract was filtered and the solvent was evaporated under vacuum at 45°C, to afford 11.4 g crude (yield 11.4%) extract. The fractionation was done according to Otsuka, 2006. Briefly, the solution was successively partitioned among *n*-hexane, CH_2_Cl_2_, ethyl acetate (EtOAc), and *n*-butanol (*n*-BuOH), and finally water. *N*-Hexane, CH_2_Cl_2_, EtOAc, *n*-BuOH, and water fractions were evaporated under vacuum to yield the residues of 0.27, 2, 0.12, trace, and 1.5 g respectively. Extracts were stored at 4°C until analysis. A partitioning scheme of *S. chorassanica *methanol extract is presented in Figure 1 (20).

All of the isolated fractions were dissolved in dimethyl sulfoxide (DMSO) and then subjected to cytotoxic and apoptosis assays.

*Cell viability*

The MTS assay (21) is based on the reduction, by mitochondrial dehydrogenase in metabolically active cells of the novel tetrazolium compound 3-(4,5-dimethylthiazol-2-yl)-5-(3-carboxymethoxyphenyl)-2-(4-sulphophenyl)-2H-tetrazolium inner salt (MTS), to the colored water-soluble formazan absorbed at 490 nm. About 5×10^4^ HeLa cells were seeded in each well of a 96-microwell plate and treated with various concentrations of each fraction of *S. chorassanica*. After incubating for 48 h, CellTiter 96^®^ Aqueous One Solution Reagent (Promega, Madison, WI, USA), which is composed of the novel tetrazolium compound MTS and an electron coupling reagent, phenazine methosulfate (PES, a redox intermediary), was added to each well according to the manufacturer’s instructions. After 3 h in culture, cell viability was determined by measuring the absorbance at 490 nm using an ELISA microplate reader (Awareness, Palm City, FL, USA). The cytotoxicity of fractions of *S. chorassanica *was expressed as IC_50_, which was calculated using Graph Pad Software (Graph Pad prism 5 software) and presented as mean ± SEM of three independent experiments with three replicates for each concentration fraction of *S. chorassanica *fractions.

*Apoptosis*

Apoptotic cells were detected using PI staining of treated cells followed by flow cytometry to detect the so-called sub-G1 peak (22, 23). It has been reported that DNA fragmentation creates small fragments of DNA that can be eluted following the incubation in a hypotonic phosphate citrate buffer. When stained with a quantitative DNA-binding dye such as PI, cells that have lost DNA will take up less stain and will appear to the left of the G1 peak. Briefly, HeLa cells were cultured overnight in a 24-well plate and treated with various concentrations of CH_2_Cl_2_ extract for 48 h. Floating cells were harvested and incubated at 4°C overnight in the dark with 750 mL of a hypotonic buffer (50 mg/mL PI in 0.1% sodium citrate+0.1% Triton X-100) before that the flow cytometric analysis using a Partec flow cytometer (GmbH, Münster, Germany) was conducted. Ten thousand events were acquired.

*Caspase activity assay*

Activity of caspases -3, -8 and -9 was measured by the colorimetric assay, using a caspase colorimetric protease kit. Cells were treated with 2 μg/mL of the CH_2_Cl_2_ fraction of *S. chorassanica*. After incubating for 48 h, the cell lysate was obtained according to the manufacturer’s instructions. The cell lysate containing 75 mg of protein was incubated with 4 mL of 4 mmole/L pNA-conjugated substrates (DEVD-pNA, IETD-pNA and LEHD-pNA; substrates for caspases -3, -8 and -9, respectively) at 37°C for 3.5 h. The amount of pNA released was measured at 405 nm using an ELISA microplate reader (Awareness, Palm City, FL, USA).

*Statistical analysis*

All results represent means ± SEM from triplicate experiments performed in a parallel manner unless otherwise indicated. Statistical analyses were performed using an unpaired, two-tailed Student’s t-test. All comparisons are made relative to the untreated controls and significance of difference is indicated as *p < 0.05 and **p < 0.01.

## Results and Discussion

*Cytotoxicity of various fractions of S. chorassanica*

**Figure 2:**
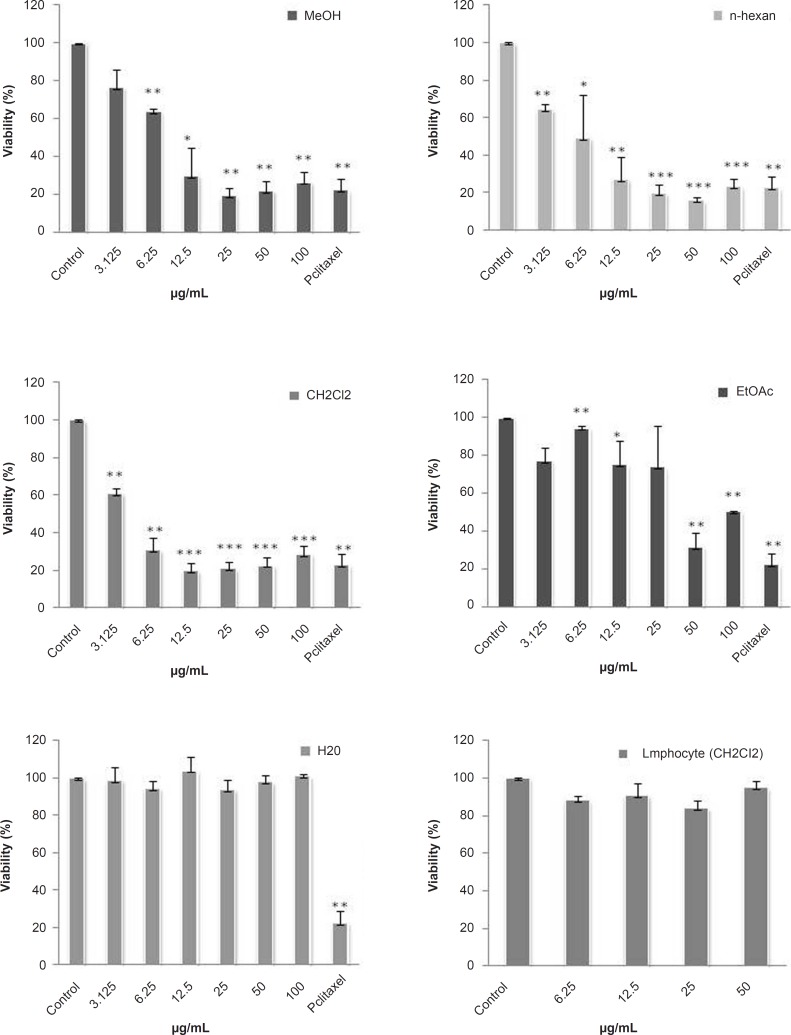
Dose-dependent growth inhibition of malignant and non-malignant cell lines by different fractions obtained from *S. chorassanica*. HeLa cells (human cervix carcinoma) were treated for 48 h in the presence of different concentrations of methanol crud extract and *n*-hexan, CH_2_Cl_2_, EtOAc, and water fractions of *S. chorassanica*. Cytotoxicity was determined by MTS assay. The CH_2_Cl_2_ fraction showed most potent inhibitory effects on the proliferation of HeLa cells. Paclitaxel was used as a positive control in a concentration of 0.35 μM. The viability of HeLa cells in this concentration was 17%. Results are the mean ± SEM of three independent experiments.

Cytotoxicity of the total methanol extract of *S. chorassanica *and its different fractions were examined on HeLa cell line. Firstly, cells were incubated with various concentrations of the total methanol extract of *S. chorassanica *(3.125-100 μg/mL) for 48 h. The results demonstrated that this extract decreased the cell viability in a concentration-dependent manner and the toxicity started at a concentration of 6.25 μg/mL (Figure 2).

In order to compare the cytotoxicity of obtained fractions of *S. chorassanica*, another MTS assay was carried out for different concentrations (3.125-100 μg/mL). Among them, the CH_2_Cl_2_ fraction was found to be more effective than the other fractions of the plant. The CH_2_Cl_2 _fraction showed most potent inhibitory effects on the proliferation of HeLa cells (Figure 2). The IC_50_ value of this fraction against HeLa cell line after 48 h was determined as 2.38 μg/mL.

IC_50_ values for different fractions in HeLa cell line are presented in Table 1.

**Table 1:** IC_50_ values (μg/mL) for different solvent fractions of *S. chorassanica *in HeLa cell line

**Cell line**	**Fraction**				
	**MeOH**	***n-*** **hexan**	**CH**_2_ **Cl**_2_	**EtOAc**	**water**	
**HeLa**	8.841	5.45	2.38	58.03	> 300	

 In comparison, the cytotoxic effect of CH_2_Cl_2_ fraction on normal lymphocyte proliferation isolated from peripheral blood was minimal (Figure 2).

 Paclitaxel was used as a positive control in a concentration of 0.35 μM. The viability of HeLa cells in this concentration was 17%.

*Apoptosis induction by the CH*_2_ *Cl*_2_ *fraction of S. chorassanica*

**Figure 3:**
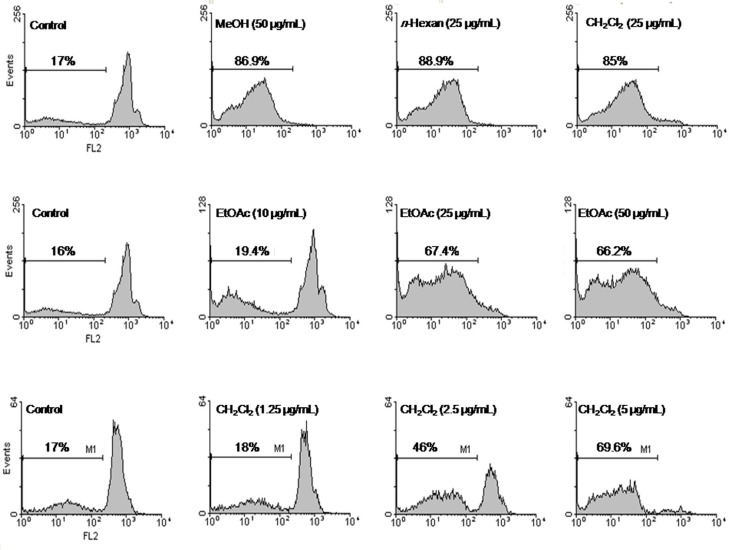
Flow cytometry histograms of apoptosis assays by PI method in HeLa cells. Cells were treated with methanol extract (50 μg/mL), *n*-hexan (25 μg/mL), and CH_2_Cl_2_ (25 μg/mL) fractions of *S. chorassanica*. Flow cytometry histograms of EtOAc-treated cells (10, 25, and 50 μg/mL) and CH_2_Cl_2_-treated cells (1.25, 2.5, and 5 μg/mL) for 48 h demonstrated concentration-dependent sub-G1 peak as an indicative of apoptotic cells, in treated but not in control cells. Results are demonstrated as the mean ± SEM of three independent experiments.

Apoptosis following the treatment with different fractions of *S. chorassanica *was measured with PI staining and flow cytometry, aiming to detect the sub-G1 peak resulting from DNA fragmentation. Flow cytometry histograms of EtOAc-treated cells (10, 25, and 50 μg/mL) and CH_2_Cl_2_-treated cells (1.25, 2.5, and 5 μg/mL) for 48 h demonstrated a concentration-dependent sub-G1 peak as an indicative of apoptotic cells, in treated but not in control cells (Figure 3).

*Caspase activity in CH*_2_ *Cl*_2_ *fraction of S. chorassanica-induced apoptosis in HeLa cells*

**Figure 4:**
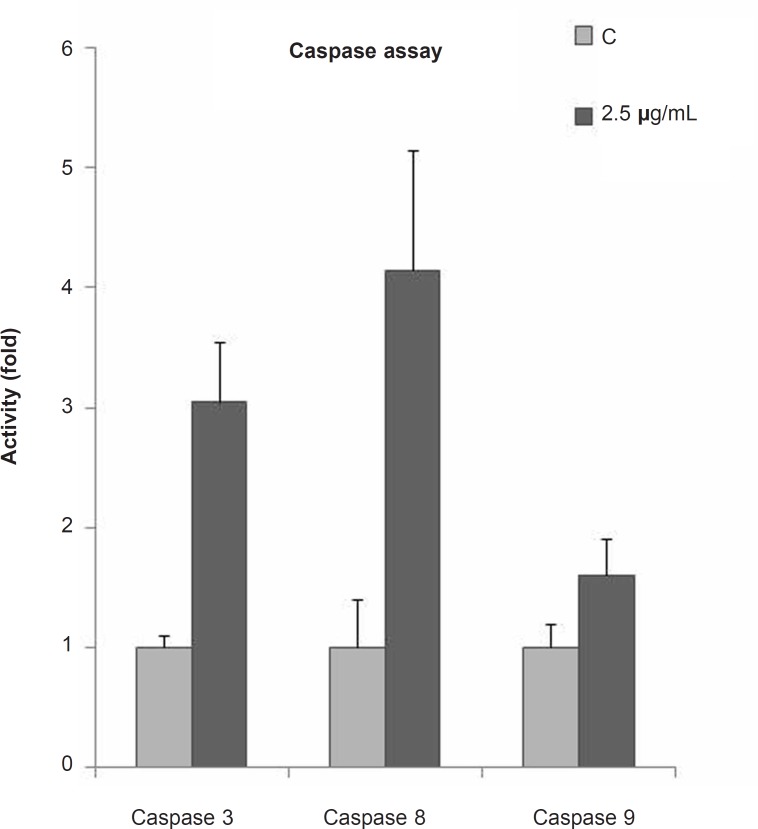
Fold activity of caspases after 48 h of treatment with 2.5 μg/mL of the CH_2_Cl_2_ fraction of *S. chorassanica *in HeLa cells. The activities of initiator caspase - 8 as well as the executioner caspase-3 were elevated significantly (p < 0.05). Results are the mean ± SEM of three independent experiments.

To examine the mechanism of the CH_2_Cl_2_ fraction of *S. chorassanica*-induced apoptosis, we measured the caspase activities using synthetic pNA-conjugated substrates. The activity of caspases, -3, -8 and -9 was evaluated after 48 h of incubation with the CH_2_Cl_2_ fraction of *S. chorassanica *(Figure 4). Finally, we observed that the activities of initiator caspase -8 as well as executioner caspase-3 were elevated significantly (p < 0.05).

This study aimed to evaluate the cytotoxic and pro-apoptotic effect of *S. chorassanica*, an Iranian endemic species of genus *Salvia *(Lamiaceae), on HeLa cell line (human cervix carcinoma).

In the first step, we examined the cytotoxicity of the crude methanol extract of *S. chorassanica *on HeLa cells by MTS assay, and confirmed that the extract possesses anti-proliferative qualities.

Many *Salvia *species have been reported to have anti-proliferative and cytotoxicity effects on several cancer cell lines (24-26). However, there was not any similar investigation on *S. chorassanica.*

Badisa *et al. *evaluated eight crude extracts of five *Salvia *species for cytotoxic activities against brine shrimps and four human cancer cell lines (HCA, HepG2, MCF-7, HPC). In the brine shrimp lethality test, all samples, except *S. fruticosa*. L. and *S. verbenaca*. L., were found to be highly active with ED_50_ values less than 300 μg/mL. In the case of human cancer cell lines, *S. fruticosa *was active against HCA cells with LC_50_ of near 50 μg/mL. Only one of the samples, *S. fruticosa*, was active against HepG2 cells with LC_50_ of 68.1 μg/mL. In the case of MCF-7 cells, *S. fruticosa *showed similar activity with LC_50_ near 40 μg/mL (27).

*In-vitro *anti-proliferative screening investigation of crude methanol extracts of six *Salvia *species including *S. dominica *L. leaves, *S. lanigera *Desf. aerial parts, *S. menthaefolia *Ten. roots, *S. palaestina *Benth. aerial parts, *S. sclarea *L. roots and *S. spinosa *L. aerial parts, revealed growth inhibitory activity with IC_50_ values ranged from 90 to 400 μg/mL (25).

Among the different *Salvia *species screened for cytotoxic activity in multitude studies, *S. chorassanica*, the plant we evaluated in this study, was shown to be the most active species with IC_50_ value of 8.841 μg/mL in HeLa cells after 48 h.

Successively, the purification by solvent extraction for the isolation of active components of *S. chorassanica *was applied and the potential antitumor activities of various fractions (*n*-hexane, CH_2_Cl_2_, EtOAc, and water-soluble) were compared. We observed that the CH_2_Cl_2_ fraction had the greatest cytotoxic effect *in-vitro*.

To determine the role of apoptosis in the cytotoxicity of *S. chorassanica*, we evaluated the percentage of apoptotic cells among cells treated with different fractions by PI staining and flow cytometry, aiming to detect the sub-G1 peak resulting from DNA fragmentation. The crude methanol extract, and specific fractions of *S. chorassanica *induced a sub-G1 peak in HeLa cells that indicates the involvement of an apoptotic process in cell death. The CH_2_Cl_2_ fraction, as the most active one, could induce apoptosis in a concentration-dependent manner compared to untreated control cells.

Apoptotic cell death is known to be induced by many chemotherapeutic agents routinely used in cancer treatment regimens. Apoptosis is characterized by distinct morphological features including chromatin condensation, cell and nuclear shrinkage, membrane blebbing and oligonucleosomal DNA fragmentation. Apoptosis is an important homeostatic mechanism that balances cell division and cell death and maintains the appropriate number of cells in the body. In the present study, apoptosis was determined using PI staining of DNA fragmentation by flow cytometry (sub-G1 peak) (28).

The induction of apoptosis in tumor cells is considered as a valuable way to treat the cancer (29). A wide variety of natural substances have been recognized to have the ability to induce apoptosis in various tumor cells. It is thus considered important to screen apoptotic inducers from plants, either in the form of crude extracts or as components isolated from them (3).

To further elucidate the mechanism of cell death induced by *S. chorassanica*, caspases-3, -8 and -9 colorimetric assays were conducted to establish the level of caspases-3 -8 and -9 activation before and after the treatment with CH_2_Cl_2_ fraction of *S. chorassanica*. Exposure of cells to the CH_2_Cl_2_ fraction of *S. chorassanica *enhanced caspase-8 activation while caspase-9 activity did not change significantly. The results of this experiment show that the treatment of HeLa cells with the CH_2_Cl_2 _fraction of *S. chorassanica *strongly increased the caspase-3 activity. This suggests the involvement of caspase-3 in triggering apoptosis in *S. chorassanica*-treated HeLa cells.

The typical executioners of apoptosis are the proteolytic enzymes called cysteinyl aspartate specific proteases. Caspases are grouped into initiator caspases (2, 8 and 10), and execution caspases (3, 6, and 7). Caspases are the essential effector molecules of apoptosis, and assaying for cleaved caspases allows detecting early apoptosis (30).

The two main pathways of apoptosis are extrinsic and intrinsic as well as a perforin/granzyme pathway. Each requires specific triggering signals to begin an energy-dependent cascade of molecular events. Each pathway activates its own initiator caspase (8, 9, 10) which in turn will activate the executioner caspase-3. Executioner caspases are common to both the extrinsic and intrinsic death pathways (31).

Caspase 8, the major extrinsic pathway protein, is the initiator of death receptor-mediated apoptosis.

Caspase-3 activation is a crucial component in the apoptotic signaling cascade. 

Based on the results obtained from our study, the apoptosis pathway involved in *S. chorassanica*-induced cell death may be through the extrinsic pathway. Further investigations are needed to clarify the exact mechanism through which *S. chorassanica *induces apoptosis. 

To sum up, this study showed the potent cytotoxic property of the crude methanol extract and various fractions of *S. chorassanica *on HeLa cell line. CH_2_Cl_2_ fraction was determined as the most cytotoxic fraction among other fractions and we observed that the cytotoxic mechanism is characterized by the induction of apoptosis. Moreover, we observed the increasing of caspases 3 and 8 activities during the apoptosis induction. 

This is the first report about the cytotoxicity and pro-apoptotic effects of *S. chorassanica*, hence further studies will be necessary to supplement our findings by fully recognizing the mechanism of cytotoxicity and cytotoxicity-conducted isolation of constituents to determine the main constituents that are responsible for the anti-proliferative effects. 
